# Identification of autophagy‐related long non‐coding RNA prognostic signature for breast cancer

**DOI:** 10.1111/jcmm.16378

**Published:** 2021-03-10

**Authors:** Qianxue Wu, Qing Li, Wenming Zhu, Xiang Zhang, Hongyuan Li

**Affiliations:** ^1^ Department of the Endocrine and Breast Surgery The First Affiliated Hospital of Chongqing Medical University Chongqing Medical University Chongqing China

**Keywords:** autophagy, breast cancer, lncRNA, prognostic, TCGA

## Abstract

Autophagy‐related long non‐coding RNAs (lncRNAs) disorders are related to the occurrence and development of breast cancer. The purpose of this study is to explore whether autophagy‐related lncRNA can predict the prognosis of breast cancer patients. The autophagy‐related lncRNAs prognostic signature was constructed by Least Absolute Shrinkage and Selection Operator (LASSO) Cox regression. We identified five autophagy‐related lncRNAs (MAPT‐AS1, LINC01871, AL122010.1, AC090912.1, AC061992.1) associated with prognostic value, and they were used to construct an autophagy‐related lncRNA prognostic signature (ALPS) model. ALPS model offered an independent prognostic value (HR = 1.664, 1.381‐2.006), where this risk score of the model was significantly related to the TNM stage, ER, PR and HER2 status in breast cancer patients. Nomogram could be utilized to predict survival for patients with breast cancer. Principal component analysis and Sankey Diagram results indicated that the distribution of five lncRNAs from the ALPS model tends to be low‐risk. Gene set enrichment analysis showed that the high‐risk group was enriched in autophagy and cancer‐related pathways, and the low‐risk group was enriched in regulatory immune‐related pathways. These results indicated that the ALPS model composed of five autophagy‐related lncRNAs could predict the prognosis of breast cancer patients.

## 1 INTRODUCTION

Breast cancer is the most common female malignant tumours, which account for a quarter of female cancer cases.^1^ Breast cancers are divided into‐Luminal A, Luminal B, HER2 enriched and basal‐like, which facilitates the adoption of precise treatment strategies and assessment of the prognosis.^2^ The prognostic variables, including PAM50 subtypes, gene expression and stromal tumour‐infiltrating lymphocytes, could be used to guide the systemic treatment of breast cancer.^3^ Therefore, researchers pay more and more attention to the role of models constructed with multiple variables in the treatment and prognostic of breast cancer patients.F

Recently, the important role of autophagy‐related lncRNA in tumours has been gradually discovered. Autophagy is a dynamic equilibrium process that degrades cellular material under cellular pressure.^4^ Lysosomes and vacuoles can locate intracellular misfolded proteins and dysfunctional organelles and degrade them to maintain the stability of the intracellular environment.^5^ Autophagy disorders are widely involved in the pathological process of various human diseases, such as cancer, neurodegeneration or immune response.^6^ Scholars are taking more care on the prognostic markers of autophagy‐related genes in different types of cancer.^7^, ^8^ Recent studies have shown that the regulation of autophagy is involved in the resistance of breast tumours to chemotherapy drugs.^9^ Moreover, long‐chain non‐coding RNA (lncRNA) is a non‐coding RNA with a length of more than 200 bp. LncRNA is widely involved in the biological behaviour of breast cancer, such as proliferation, apoptosis, invasion and metastasis.^10^, ^11^, ^12^ Interestingly, lncRNA also plays a vital role in regulating autophagy.^13^ Studies have shown that lncRNA‐mediated autophagy phenomenon plays an important role in breast cancer resistant to tamoxifen or trastuzuma.^14^, ^15^ On the other hand, increasing evidence has been presented that the use of autophagy‐related lncRNAs to predict tumour patients outcomes.^16^

In this study, we hypothesized that a variable model composed of multiple autophagy‐related lncRNAs could be used to predict the prognosis of breast cancer patients. The lncRNA, mRNA expression dataset and clinical pathological features of breast cancer from The Cancer Genome Atlas (TCGA), were used to assess prognostic value of autophagy‐related lncRNAs. Finally, we employed an autophagy‐related lncRNA prognostic signature (ALPS) model to effectively predict the prognosis of breast cancer patients.

## 2 METHODS

### 2.1 Patient data sets

RNA‐seq expression and clinical information of 1,108 breast cancer patients were obtained from The Cancer Genome Atlas (TCGA) data portal (https://cancergenome.nih.gov/). Ensembl human genome browser, GRH38.p13 (http://asia.ensembl.org/index.html), was used to annotate and classify 14,142 lncRNAs and 19,658 protein‐coding genes. Male subjects or patients with less than 30 days overall survival (OS) were excluded, 1,027 breast cancer patients were used in the present study. The patients were randomly divided into a training and testing group. After excluding patients with incomplete clinical pathological data, this study enrolled 569 patients for subsequent analysis.

### 2.2 Identification of autophagy‐related lncRNAs in breast cancer

232 autophagy‐related genes come from the Human Autophagy Database (HADB; http://www.autophagy.lu/index.html). Moussay et al detailed descriptions of human autophagy‐related genes.^17^ The Pearson correlation coefficient method was used to screen autophagy‐related lncRNAs with |R|>0.3 and *P* < 0.001.

### 2.3 Construction of autophagy‐related lncRNA prognostic signatures for breast cancer

The univariate Cox regression model was used to analyse the relationship between the expression level of autophagy‐related lncRNA and the OS in breast cancer patients (*P* < 0.05). A Least Absolute Shrinkage and Selection Operator (LASSO) Cox regression analysis of prognostic‐related autophagy‐associated lncRNAs using the ‘glmnet’ package was performed in R software. To evaluate its independent prognostic effect on survival, multivariate Cox regression analysis was used to analyse autophagy‐related lncRNAs candidates. Therefore, an ALPS model composed of five autophagy‐related lncRNAs was constructed. This ALPS model selects the best lncRNA prognostic markers based on the lowest Akaike information criterion (AIC) value. The risk score of each patient was calculated according to the following formula: Risk Score = $\sum_{k=0}^{n}\text{coef}\left(k\right)*x\left(k\right)$, where coef(k) and *x*(*k*) represent regression coefficient and the expressive value of each autophagy‐related lncRNA, respectively.^18^

### 2.4 Independent prognostic analysis and ROC curve plotting

The Kaplan‐Meier survival curve and log‐rank test were used to compare the OS of the high‐risk group and the low‐risk group. The cut‐off value of the risk score was employed to divide patients into high and low‐risk groups. Cox proportional risk modelling was fitted to estimate crude and multivariable‐adjusted hazard ratios (HRs) and 95% confidence intervals (CI), and potential covariates involved age, TNM stage, tumour size (T), lymph node metastasis (N), distant metastasis (M), risk score, ER status, PR status and HER2 status. The accuracy of each clinicopathological feature and risk score in predicting survival time was evaluated by the receiver operating characteristic (ROC) curve.

### 2.5 Nomogram

Nomogram was utilized to predict the probable 1‐year, 3‐year and 5‐year survival of breast cancer patients. A nomogram was constructed by integrating with clinical pathological variables such as age, stage, T stage, N stage, M stage, ER status, PR status, HER2 status and the risk score derived from the prognostic signature.

### 2.6 Principal component analysis (PCA) and Gene set enrichment analysis (GSEA)

PCA was used to investigate the distribution of patients with different risk score. GSEA version 4.0.3 (Broad Institute, USA) was used to analyse the genes that were differentially expressed between the high‐ and low‐risk group patients. 1000 permutations were selected, and Affymetrix was used as the chip platform for the calculation of the normalized enrichment score (NES). Normal *P*‐value < 0.05 and false discovery rate (FDR q‐value) <0.25 were considered significantly enriched.^19^

### 2.7 Construction of the LncRNA‐mRNA co‐expression network

The correlation between autophagy‐related lncRNA and its co‐expressed mRNA was analysed by co‐expression network and Sankey Diagram. Cytoscape software (version 3.7.1, http://www.cytoscape.org/) and ggalluvial R package were used to visualize the co‐expression network and Sankey Diagram.^20^

### 2.8 Statistical analysis

The statistical analysis of all data was performed using R software (version 4.0.3, https://www.r‐project.org/). *P* < 0.05 was regarded as statistically significant.

## 3 RESULTS

### 3.1 Identification of prognostically significant autophagy‐related lncRNAs in breast cancer patient tissue samples

**FIGURE 1:**
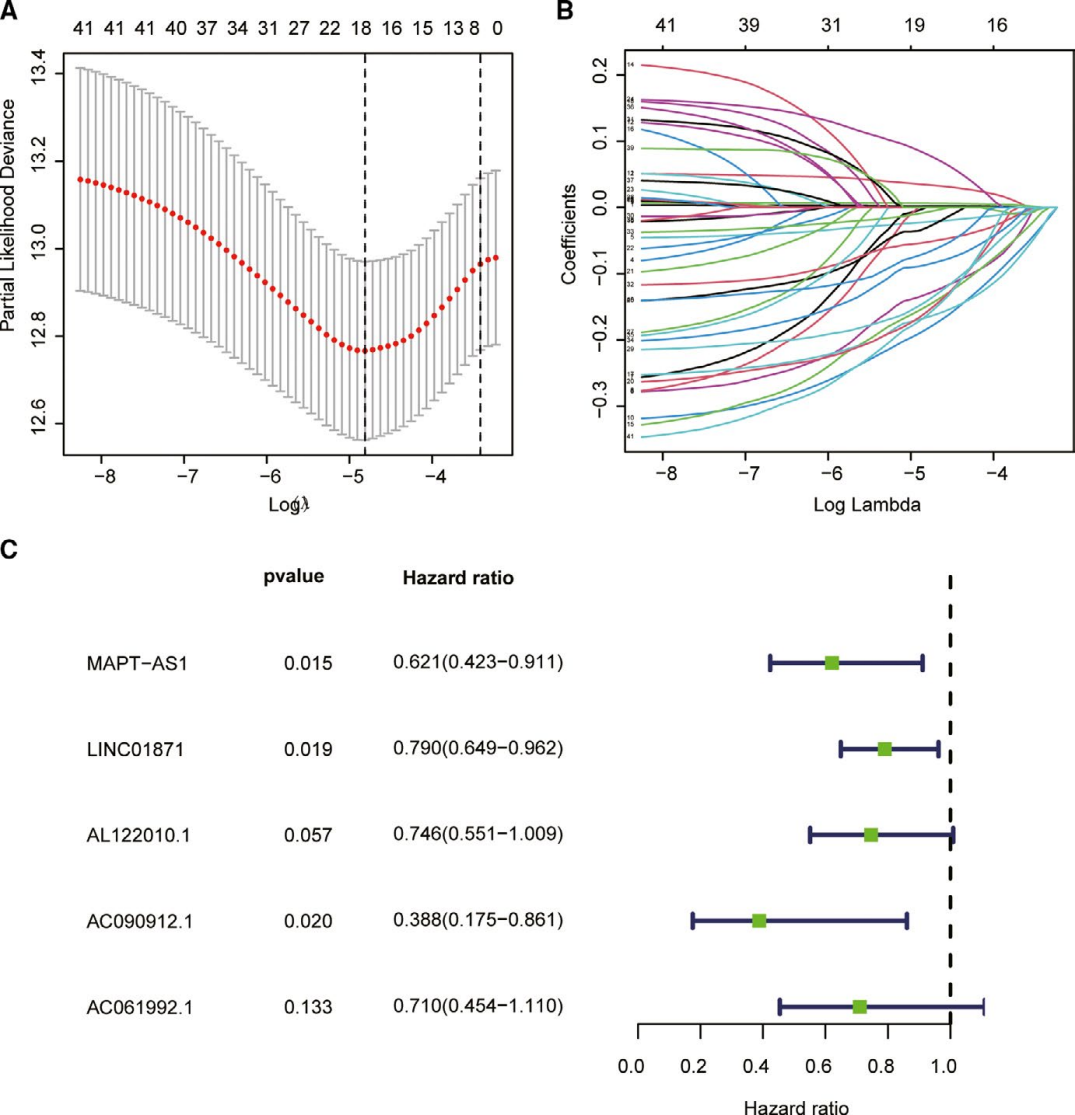
Autophagy‐related lncRNA selection utilizing Lasso model. A, Plots of the ten‐fold cross‐validation error rates. B, LASSO coefficient profiles of the five autophagy‐related lncRNAs. C, The univariate Cox regression analysis results show that 5 autophagy‐related lncRNAs

1,270 autophagy‐related lncRNAs were identified from 14,142 lncRNAs and 232 autophagy‐related genes via the criterion with |*R*|>0.3 and *P* < 0.001. Univariate Cox proportional hazard analysis showed that 41 autophagy‐related lncRNAs were significantly related to the survival of breast cancer patients (Table S1). In the training group, LASSO Cox regression was used to screen prognostic autophagy‐related lncRNAs based on 1,000 times ten‐fold cross‐validation (Figure 1A,B). Multivariate Cox analysis further ascertained five lncRNAs with prognostic significance, namely MAPT‐AS1, LINC01871, AL122010.1, AC090912.1, AC061992.1. These five lncRNAs were employed to construct an ALPS model (Figure 1C).

### 3.2 Evaluation of the ALPS model consisting of five autophagy‐related lncRNAs

**FIGURE 2:**
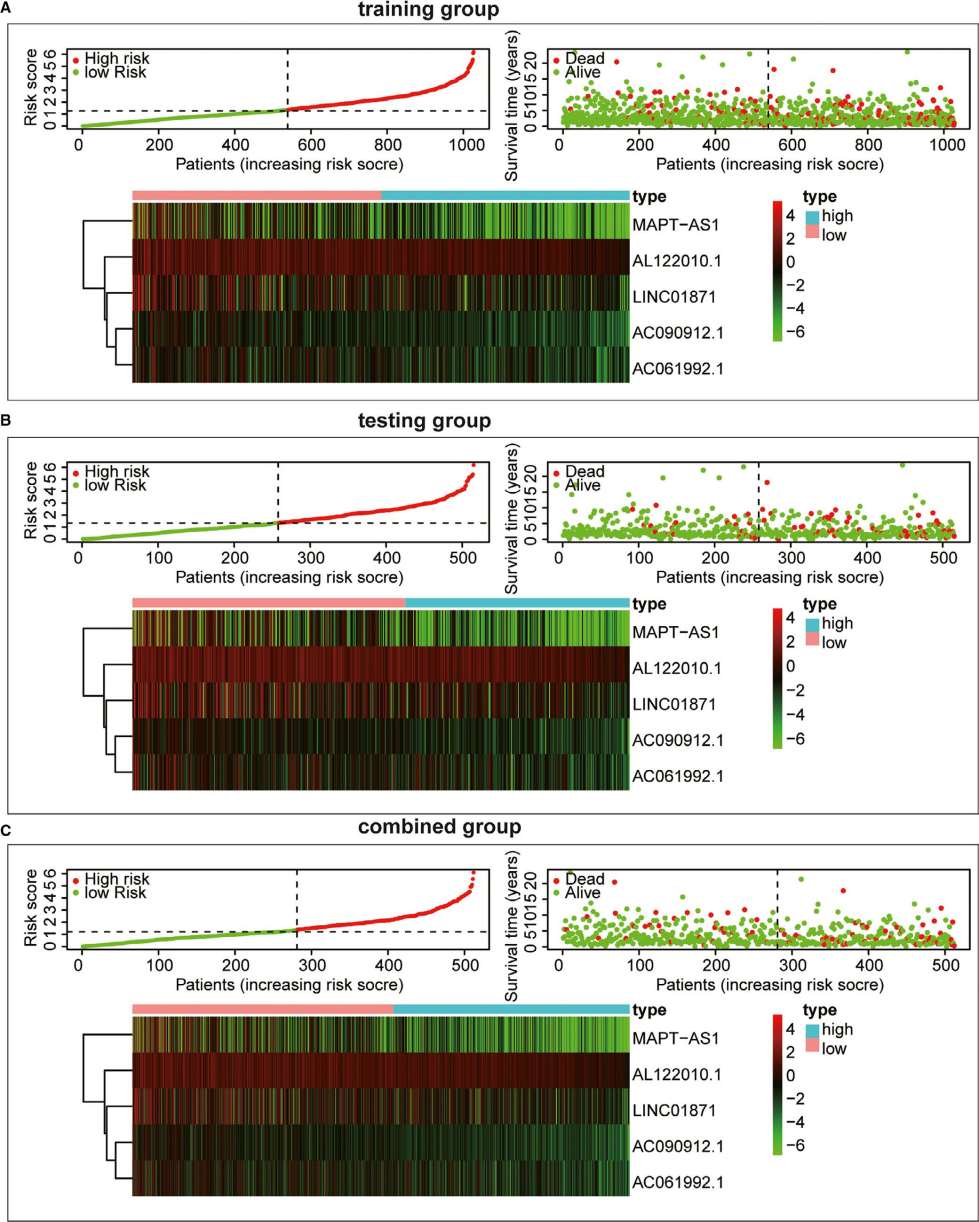
Construction and validation of the ALPS in the three groups. A, Distribution of BRCA patients and Survival status of BRCA patients with different risk scores, and Heatmap of the lncRNA signature in the training group. B, Distribution of BRCA patients and Survival status of BRCA patients with different risk scores, and Heatmap of the lncRNA signature in the testing group. C, Distribution of BRCA patients and Survival status of BRCA patients with different risk scores, and Heatmap of the lncRNA signature in the combined group

**FIGURE 3:**
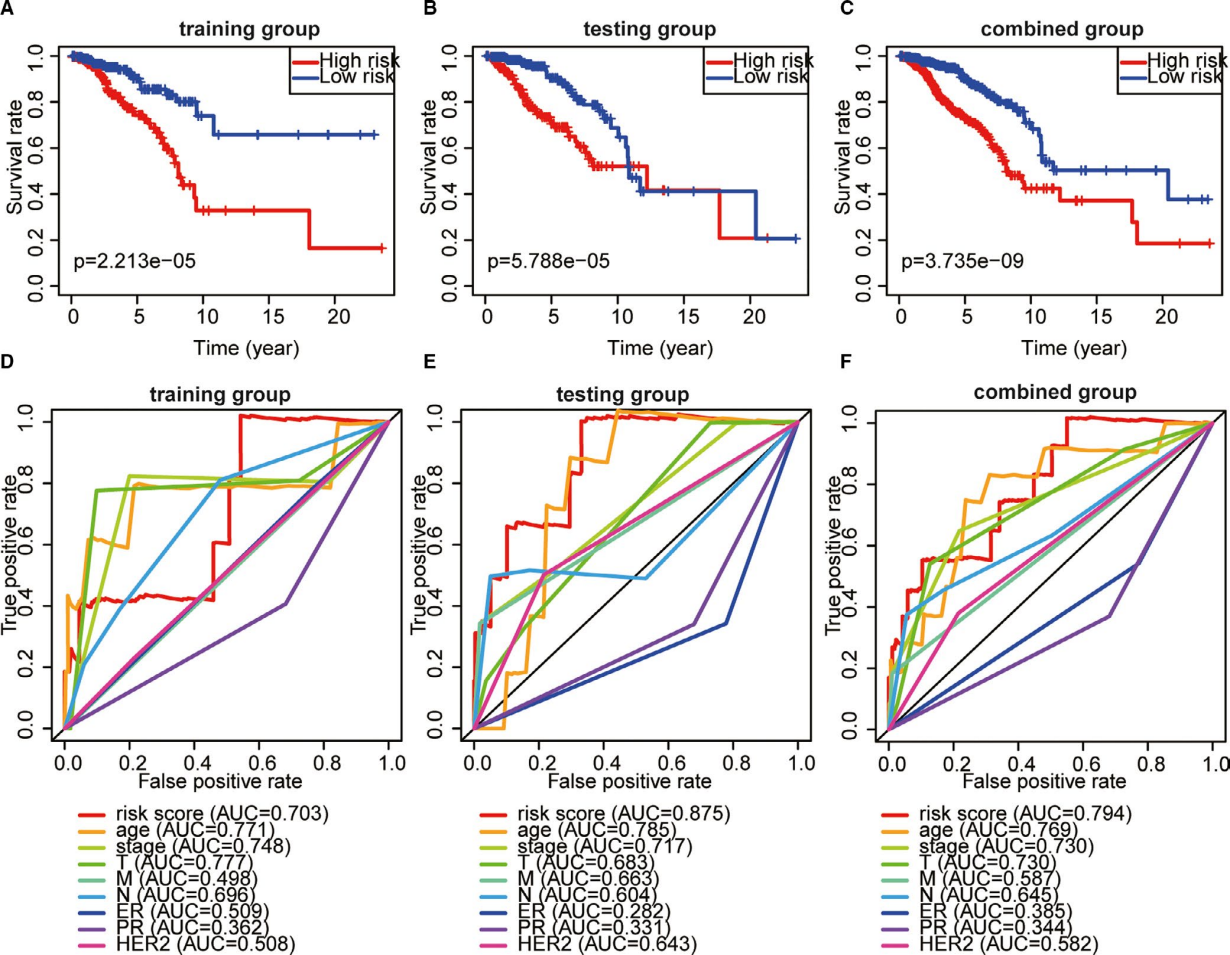
Prognostic significance analysis of the ALPS. A‐C, Kaplan‐Meier survival curve analysis shows that survival time of patients with high‐risk scores based on the autophagy‐related lncRNA prognostic signature is significantly shorter than those with low‐risk scores in the training group, testing group, and combined group. D‐F, The AUC for risk model score and clinical features according to the ROC curves in the training group, testing group, and combined group. Clinical feature: Age, stage, and T, N, M stage, and ER, PR, HER2 status

According to median value of risk score based on ALPS model, breast cancer patients were divided into high‐risk groups and low‐risk groups. Draw risk curves and scatter plots were used to illustrate the risk score and corresponding survival status of breast cancer patients. The results showed that the higher the risk score, the higher the mortality rate was observed in the training group, test group and combined group, respectively (Figure 2). The heatmap also showed that MAPT‐AS1, LINC01871, AL122010.1, AC090912.1, AC061992.1 were up‐regulated in low‐risk breast cancer (Figure 2). Kaplan‐Meier survival analysis showed that the OS of the high‐risk group was inferior than those of the low‐risk group (Figure 3A‐C; *P* < 0.001). ROC curve analysis showed that the AUC value of the risk score based on ALPS model was greater than 0.7, which was greater than most other clinical prognostic indicators, such as stage and T stage, N stage, M stage, ER status, PR status and HER2 status (Figure 3D‐F).

### 3.3 Correlation of the risk score of ALPS model with clinicopathological factors

**FIGURE 4:**
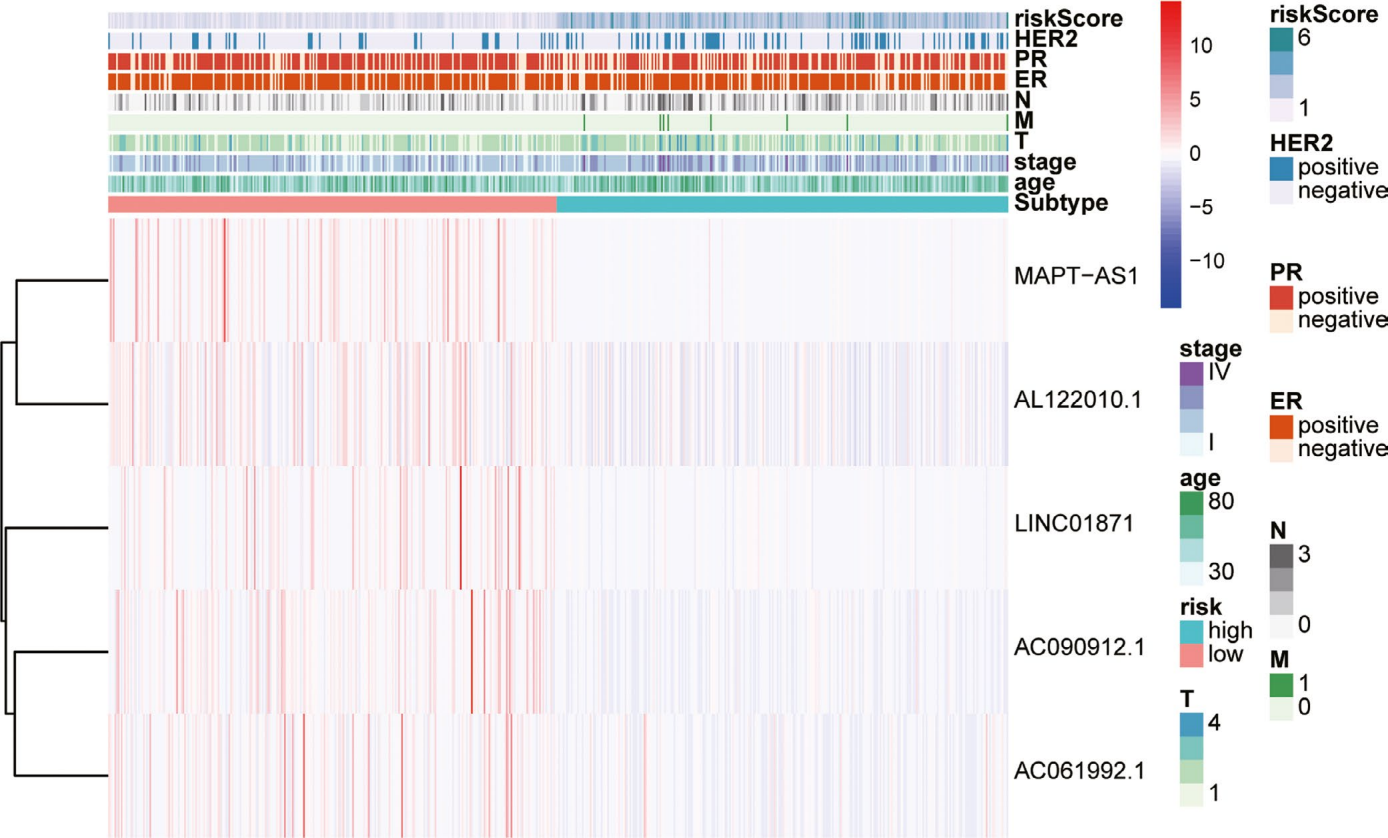
Relationship between the risk score and clinical significance

**FIGURE 5:**
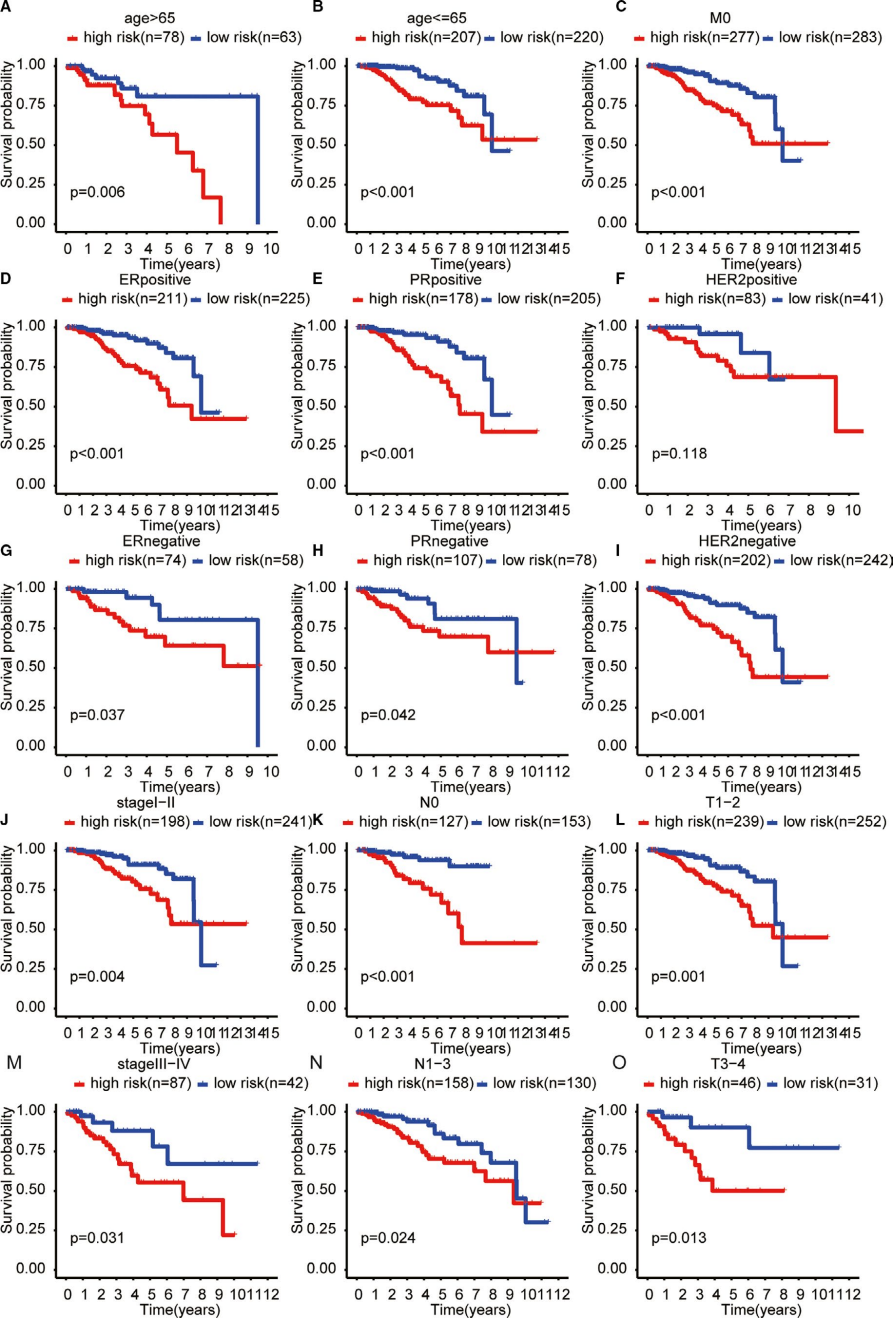
Subgroup analysis of the ALPS for (A) age > 65 y; (B) age < 65 y; (C) M0;(D) ER positive;(E) PR positive;(F) HER2 positive;(G) ER negative;(H) PR negative;(I) HER2 negative;(J) stage I‐II;(K) NO;(L) T1‐2;(M) stage III‐IV;(N) N1‐3;(O) T3‐4

To further explore whether the ALPS model was associated with the characteristics of breast cancer, we evaluated the relationship between the risk score of ALPS model and clinical characteristics. The risk score of ALPS model was significantly correlated with Stage, ER status, PR status and HER2 status (Table 1): risk score of stage III‐IV is significantly higher than stage I‐II’s (*P* < 0.001), and risk score of T3‐4 is significantly higher thanT‐2’s (*P* = 0.01), etc Figure 4 showed that the risk score of ALPS model differed according to clinicopathological features and tumour. And the difference of five lncRNA signature score based on different molecular subtypes showed in Figure S1. In addition, subgroup survival analysis showed that the overall survival of the high‐risk group based on the ALPS model was significantly worse than those of the low‐risk group (except for the HER2 subgroup) (Figure 5A‐O; *P* < 0.05). Figure S2 visualized the co‐expression network of five autophagy‐related lncRNAs and their regulated mRNAs from the ALPS model.

**TABLE 1:** The relationship of breast cancer patients clinical feature and the ALPS model

Clinical	Group	*n*	Mean	*SD*	*t*	*P*
age	<=65	427	1.552	1.13	‐1.24938	0.213
	>65	141	1.687	1.106		
stage	Stage I‐II	439	1.466	1.063	‐4.41758	0
	Stage III‐IV	129	1.994	1.231		
T	T1‐2	491	1.53	1.086	‐2.61782	0.01
	T3‐4	77	1.938	1.297		
M	M0	560	1.573	1.114	‐1.68429	0.135
	M1	8	2.498	1.548		
N	N0	280	1.474	1.075	‐2.34069	0.02
	N1‐3	288	1.694	1.162		
ER	negative	132	1.816	1.285	2.447189	0.015
	positive	436	1.516	1.063		
PR	negative	185	1.835	1.235	3.507381	0.001
	positive	383	1.465	1.048		
HER2	negative	444	1.438	1.031	‐5.42613	0
	positive	124	2.115	1.279		

Abbreviations: ER, oestrogen receptor; HER2, human epidermal growth factor receptor‐2; M, distant metastasis, stage according to AJCC 8th classification; N, lymph node; PR, progesterone receptor; T, tumour size

### 3.4 The ALPS model is an independent prognostic factor for patients with breast cancer

**FIGURE 6:**
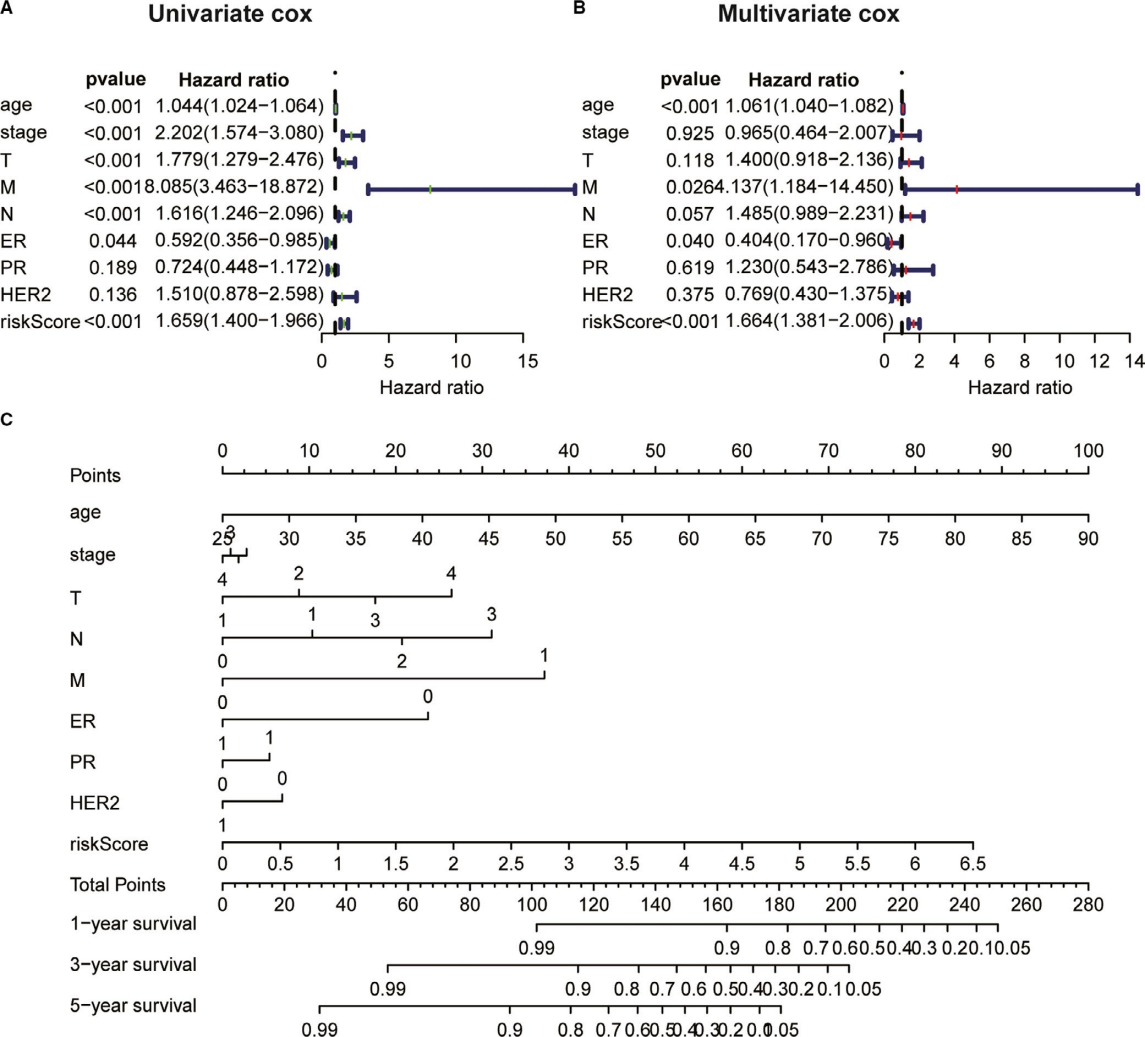
A, The univariate and B, multivariate Cox regression analysis of risk model score and clinical feature prognostic value. C, Nomogram used to predict prognosis in patients with cervical cancer at 1, 3, and 5 years based on risk score, age, TNM stage, and ER, PR, HER2 status

Next, we performed univariate and multivariate Cox regression analyses to determine that ALPS model could be used as an independent risk factor for breast cancer patients. Multivariate Cox regression analysis showed that age (HR = 1.061, 1.040‐1.082, *P* < 0.001) and the risk score of ALPS model (HR = 1.664, 1.381‐2.006, *P* < 0.001) were independently associated with OS (Figure 6B). These data indicated that the ALPS model was an independent prognostic factor in breast cancer patients. A nomogram map was performed to predict 1‐, 3‐ and 5‐year survival in breast cancer patients using stage, T stage, M stage, N stage, ER status, PR status, HER2 status and risk score (Figure 6C).

### 3.5 Principal component analysis and Gene set enrichment analysis

**FIGURE 7:**
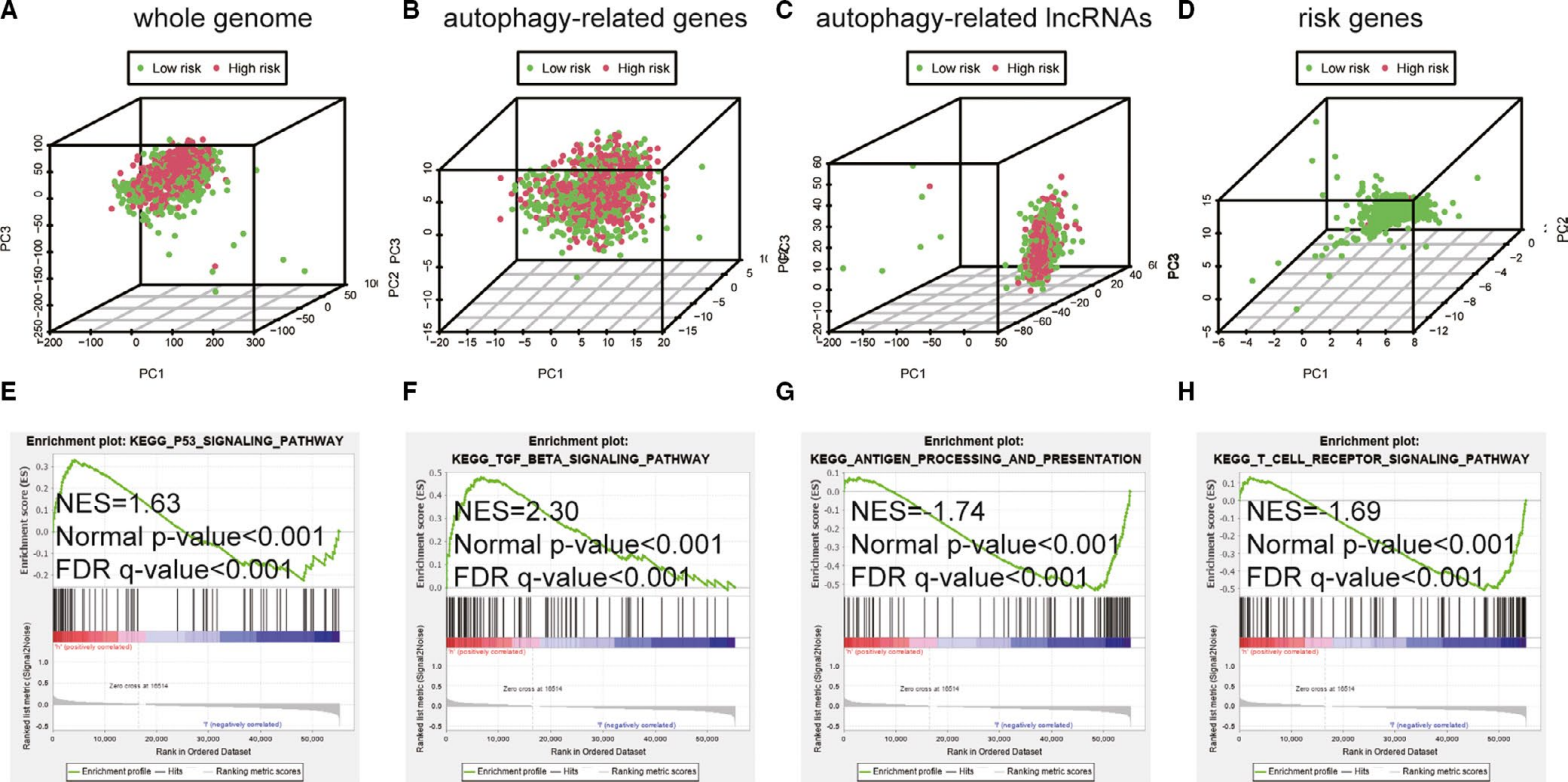
Patients with high and low‐risk scores have different autophagy statuses. PCA maps show the distribution of patients based on the A, whole genome; B, autophagy‐related gene sets; C, autophagy‐related lncRNAs; and D, ALPS. E‐H, Functional enrichment analysis based on the ALPS model by GSEA. Significantly enriched KEGG pathways and oncogenic signatures in the high‐risk groups

We performed PCA maps to visualize the distribution of patients based on the whole genome, autophagy‐related gene sets, autophagy‐related lncRNAs and five lncRNAs from the ALPS model (Figure 7A‐D). The results showed that, different from other gene sets, the five lncRNAs from ALPS model tend to be low‐risk distribution. GSEA results showed that the genes enriched in high‐risk breast cancer patients were related to positive regulation of TGF‐beta signalling pathway, P53 signalling pathway (Figure 7E, F). Anti‐cancer immunomodulatory pathways were significantly up‐regulated in the low‐risk group, including pathways related to antigen processing and presentation, T cell receptor signal transduction (Figure 7G, H).

## 4 DISCUSSION

In this study based on autophagy‐related lncRNAs and clinical data from TCGA, we found that an autophagy‐related lncRNA prognostic signature (ALPS) model could well predict prognosis of breast cancer patients. Notably, the predictive function of ALPS model was independent of other clinical pathological features.

Currently, the most valuable prognostic factors of breast cancer were included Nottingham Prognostic Index (NPI), and pathological features of stage, ER, PR, HER2, while the treatment strategy of patients depends on their pathological characteristics.^21^, ^22^ However, due to the limitations of traditional clinicopathological features, the clinical outcome of breast cancer patients is still highly heterogeneous.^23^ Autophagy has been reported to play an important role in the progression and recurrence of cancer.^24^ In addition, increasing evidence showed that lncRNA plays a crucial role in regulating autophagy in various cancers, such as breast cancer,^16^ endometrial cancer,^25^ liver cancer^26^ and lung cancer.^27^ Thus, autophagy‐related lncRNAs have important diagnostic and prognostic implications.

In our present study, we identified an ALPS model composed of five autophagy‐related lncRNAs from the TCGA dataset. Our results suggested that the ALPS model is a clinical valuable prognostic biomarker in breast cancer. Specifically, the risk score of ALPS model could divide the population of breast cancer into two prognostically distinct groups. To accomplish this, the assay integrates five lncRNAs and presented a single prognostic score as a continuous variable and proposes specific cut‐offs (risk score = 1.3528). Importantly, a nomogram that was integrated with multiple variables (including the risk score) can predict the survival of breast cancer patients. Finally, an intriguing finding was that the distribution of five lncRNAs from the ALPS model tend to be low‐risk, which was consistent with other studies. GSEA analysis also showed that based on the ALPS model, the high‐risk group was enriched in tumour‐related pathways, while the low‐risk group was positively correlated with immune function pathways.

Of the five autophagy‐related lncRNAs in the ALPS model, only MAPT‐AS1, LINC0187 and AL122010.1 have been studied in breast cancer or other cancers. MAPT‐AS1 is reported as an independent prognostic marker of clear cell renal cell carcinoma (ccRCC), inhibiting the proliferation and invasion of ccRCC.^28^ Likewise, Wang et al also reported MAPT‐AS1 being a kind of lncRNA, which exists in the antisense chain of microtubule‐associated protein tau promoter. MAPT‐AS1 up‐regulation is related to the better survival of breast cancer patients.^29^ However, it was reported that lncRNA MAPT‐AS1 promotes the proliferation and migration of breast tumour cells through antisense pairing with MAPT, reducing the sensitivity of cancer cells to paclitaxel. The possible reason for this contradiction is that the role of MAPT‐AS1 in different types of breast cancer patients is inconsistent. LINC01871 is involved in the construction of an immune prognostic model of gastric cancer.^30^ Consistent with the results of our study, LINC01871 and AL122010.1 tended to be low‐risk factors for participate in the construction of a stemness‑related prognostic model of breast cancer.^18^ In this study, the co‐expression network composed of lncRNA and mRNA was constructed to predict the possible functions of the lncRNA from the mRNA with known biological functions (Figure S2). For example, in this study, FAS and CASP1 co‐expressed with LINC01871 are associated with promoting cell apoptosis.^31^, ^32^ We can speculate that LINC01871 may be a protective factor in breast cancer.

The advantage of our study is that a small amount of lncRNA (only five lncRNA) can well predict the survival of breast cancer patients, so they can be better applied in clinical practice. Taken together, relevant research reports of these five lncRNAs further conform that our research direction is promising. This study also has several limitations. Firstly, that only TCGA dataset was used to internally validate the predictive accuracy of ALPS model but lacked cross‐validation externallyNeither METABRIC dataset nor GEO dataset obtained these five lncRNAs spontaneously. Secondly, the predict benefits of endocrine therapy and chemotherapy were not explored in ALPS model. Consequently, in the future research, it may be necessary to recruit cohorts of breast cancer patients (or drug‐resistant patients) to validate the predictive accuracy of the ALPS model, especially for predicting benefits of endocrine therapy and chemotherapy.

In conclusion, development of ALPS model integrating five lncRNAs (MAPT‐AS1, LINC01871, AL122010.1, AC090912.1, AC061992.1), derived from machine learning based screening, could well predict the survival of breast cancer patients. Future prospective clinical trials are needed to further consolidate the effectiveness of the ALPS model.

## CONFLICT OF INTEREST

The authors declare that they have no competing interests.

## AUTHOR CONTRIBUTION

**Qianxue Wu:** Conceptualization (lead); Data curation (lead); Formal analysis (lead); Methodology (lead); Writing‐original draft (lead). **Qing Li:** Data curation (equal); Formal analysis (supporting). **Xiang Zhang:** Supervision (equal); Writing‐review & editing (equal). **Wenming Zhu:** Data curation (equal); Formal analysis (supporting). **Hong‐Yuan Li:** Supervision (lead); Writing‐review & editing (supporting).

## Supporting information

Fig S1

Fig S2

Table S1

## Data Availability

All data utilized in this study are included in this article, and all data supporting the findings of this study are available on reasonable request from the corresponding author.
