# Supplementary figures and images for: Identification of autophagy‐related long non‐coding RNA prognostic signature for breast cancer
Source: J Cell Mol Med. 2021 Mar 10;25(8):4088–98. doi: 10.1111/jcmm.16378 (PMC8051719; doi:10.1111/jcmm.16378)

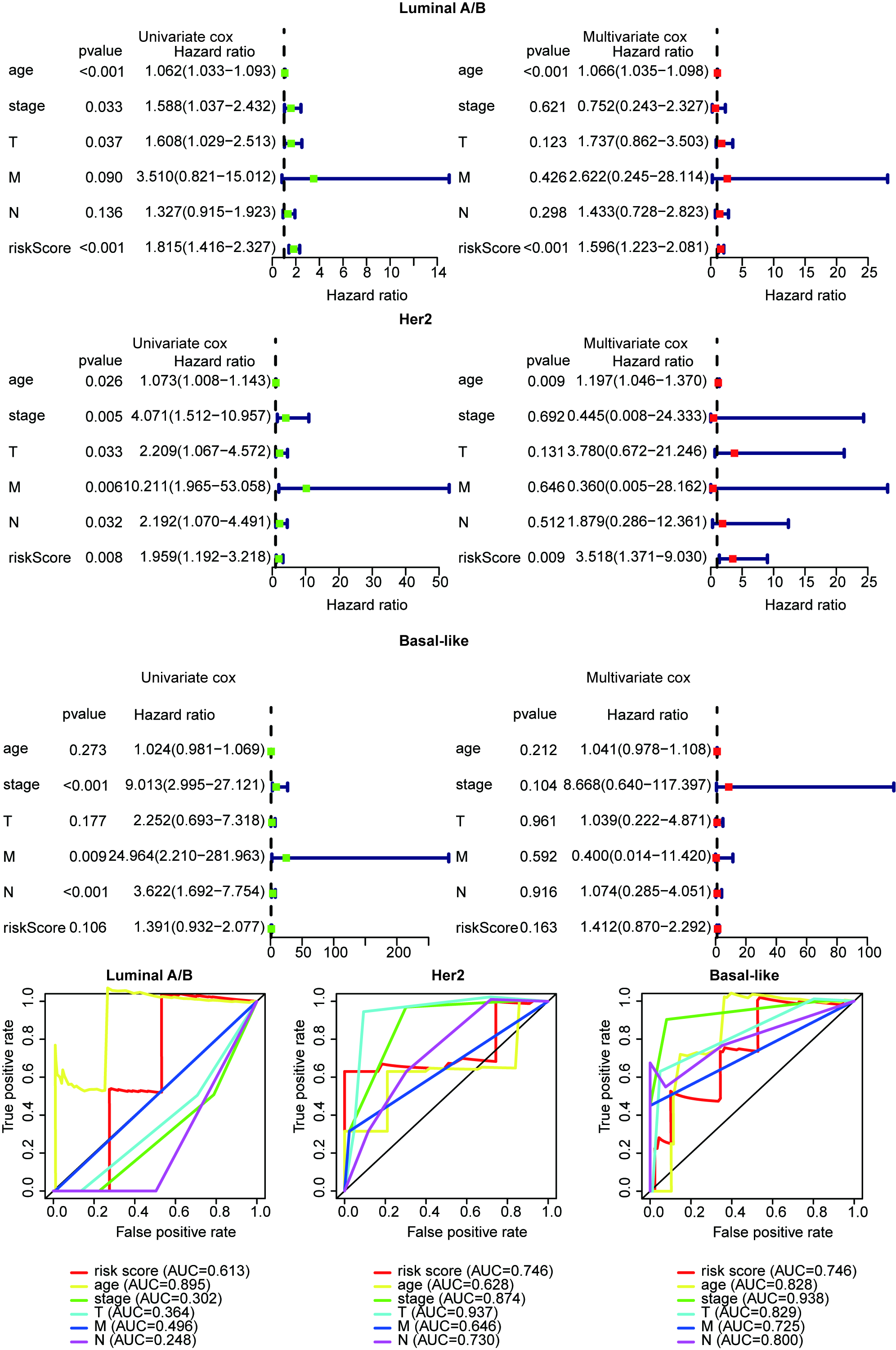

Supplement: Supplementary file 1 — Fig S1 [file JCMM-25-4088-s002.tif]

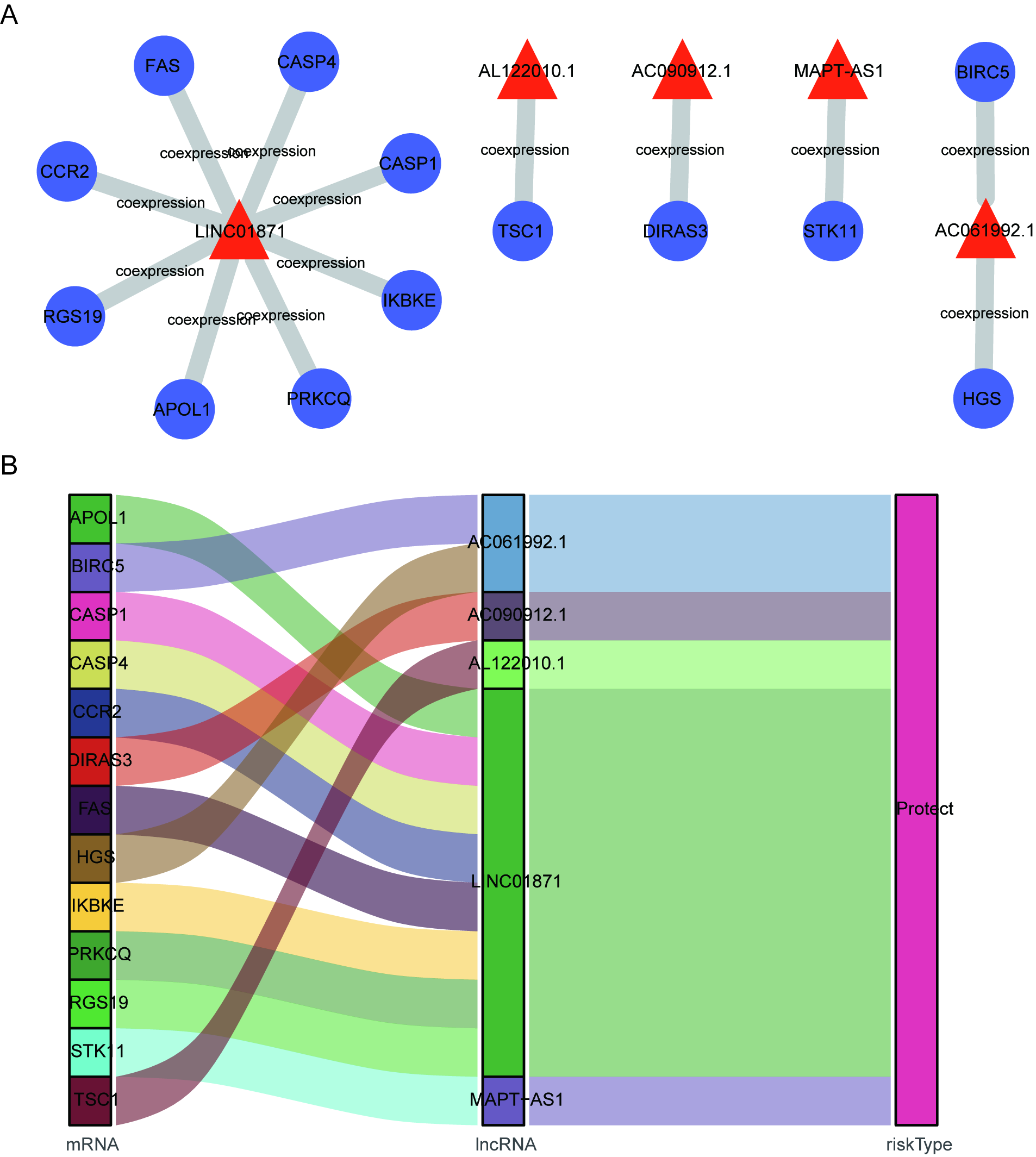

Supplement: Supplementary file 2 — Fig S2 [file JCMM-25-4088-s003.tif]
