# Supplementary material for: Identification of autophagy‐related long non‐coding RNA prognostic signature for breast cancer
Source: J Cell Mol Med. 2021 Mar 10;25(8):4088–98. doi: 10.1111/jcmm.16378 (PMC8051719; doi:10.1111/jcmm.16378)
Supplement: Supplementary file 3 — Table S1 [file JCMM-25-4088-s001.docx]

Table S1 Univariate cox proportional hazard analysis showed that 41 autophagy-related lncRNAs were significantly related to the survival of TCGA breast cancer

| gene | KM | B | SE | HR | HR.95L | HR.95H | P value |
| --- | --- | --- | --- | --- | --- | --- | --- |
| LINC01614 | 0.025949 | 0.0212 | 0.007799 | 1.021426 | 1.005931 | 1.03716 | 0.006564 |
| OTUD6B-AS1 | 0.023584 | 0.077714 | 0.026502 | 1.080814 | 1.026107 | 1.138437 | 0.003363 |
| AC103691.1 | 0.022084 | -0.20348 | 0.098169 | 0.815888 | 0.673083 | 0.988991 | 0.038197 |
| AC020907.4 | 0.010011 | -0.30646 | 0.146907 | 0.736045 | 0.551896 | 0.98164 | 0.036969 |
| SEMA3B-AS1 | 0.025546 | -0.07632 | 0.030083 | 0.926521 | 0.873471 | 0.982792 | 0.011182 |
| ST7-AS1 | 0.002785 | -0.64373 | 0.194123 | 0.525327 | 0.359081 | 0.768542 | 0.000913 |
| AC024361.1 | 0.001985 | -0.47607 | 0.211436 | 0.621222 | 0.410462 | 0.940203 | 0.024349 |
| AL136531.1 | 0.015276 | -0.40701 | 0.200373 | 0.665638 | 0.449448 | 0.985816 | 0.042229 |
| LINC01235 | 0.013683 | 0.011099 | 0.00462 | 1.011161 | 1.002047 | 1.020358 | 0.01628 |
| MAPT-AS1 | 0.001782 | -0.31088 | 0.097333 | 0.7328 | 0.60553 | 0.886819 | 0.001403 |
| AC087741.1 | 0.002537 | -0.22374 | 0.097376 | 0.79952 | 0.660606 | 0.967644 | 0.021577 |
| NIFK-AS1 | 0.001127 | -0.42672 | 0.147582 | 0.652646 | 0.488715 | 0.871565 | 0.003835 |
| AC107464.3 | 0.008343 | -0.19361 | 0.074239 | 0.823977 | 0.712398 | 0.953032 | 0.009108 |
| AC005840.4 | 0.034327 | -0.33413 | 0.162455 | 0.715964 | 0.520726 | 0.984403 | 0.039712 |
| LINC01871 | 0.030842 | -0.21012 | 0.063933 | 0.810484 | 0.715031 | 0.91868 | 0.001014 |
| AC010201.2 | 0.002428 | -0.53003 | 0.240961 | 0.588588 | 0.367033 | 0.943883 | 0.027832 |
| AL122010.1 | 0.004509 | -0.34274 | 0.097048 | 0.709825 | 0.586872 | 0.858536 | 0.000413 |
| STAG3L5P-PVRIG2P-PILRB | 0.009017 | -0.36683 | 0.158268 | 0.692926 | 0.508123 | 0.94494 | 0.02046 |
| AC234582.1 | 0.003214 | -0.27207 | 0.12391 | 0.761804 | 0.597546 | 0.971216 | 0.028116 |
| AC090912.1 | 0.029827 | -0.66741 | 0.256931 | 0.513036 | 0.310061 | 0.848883 | 0.009387 |
| PRKCZ-AS1 | 0.045006 | -0.37768 | 0.144073 | 0.685448 | 0.516821 | 0.909095 | 0.008755 |
| AL451085.2 | 0.021909 | -0.41206 | 0.16024 | 0.662284 | 0.48378 | 0.906652 | 0.010125 |
| USP30-AS1 | 0.008837 | -0.21671 | 0.085407 | 0.805162 | 0.681059 | 0.951878 | 0.011168 |
| DNAH10OS | 0.035954 | 0.132864 | 0.067426 | 1.142094 | 1.000711 | 1.303452 | 0.04878 |
| AL031186.1 | 0.023566 | -0.33976 | 0.16926 | 0.711938 | 0.510937 | 0.992012 | 0.044713 |
| AC136475.2 | 0.008706 | -0.17851 | 0.080488 | 0.836512 | 0.714432 | 0.979452 | 0.026561 |
| TNFRSF14-AS1 | 0.000489 | -0.60356 | 0.200199 | 0.546863 | 0.369376 | 0.809633 | 0.002572 |
| SH3BP5-AS1 | 0.002814 | -0.26275 | 0.102611 | 0.768936 | 0.62885 | 0.940228 | 0.010449 |
| AC061992.1 | 0.032123 | -0.33308 | 0.137249 | 0.716716 | 0.547672 | 0.937936 | 0.015232 |
| FLJ42351 | 0.01571 | -0.52236 | 0.219701 | 0.593116 | 0.385594 | 0.912325 | 0.017425 |
| AL109811.3 | 0.026775 | -0.16502 | 0.070826 | 0.847881 | 0.737985 | 0.974141 | 0.019813 |
| AL358472.3 | 0.034949 | -0.29422 | 0.10978 | 0.745113 | 0.600865 | 0.923989 | 0.00736 |
| EGOT | 0.004501 | -0.13464 | 0.063131 | 0.874029 | 0.772304 | 0.989152 | 0.032946 |
| AC121761.2 | 0.000515 | -0.56768 | 0.218906 | 0.56684 | 0.369085 | 0.870549 | 0.009507 |
| AC004067.1 | 0.034951 | -0.37073 | 0.185711 | 0.690228 | 0.47964 | 0.993276 | 0.045902 |
| LINC01786 | 0.028052 | -0.28703 | 0.132963 | 0.750489 | 0.578318 | 0.973919 | 0.030872 |
| PCED1B-AS1 | 0.007146 | -0.10136 | 0.048155 | 0.903603 | 0.82222 | 0.993042 | 0.035295 |
| AP005131.2 | 0.006048 | -0.33345 | 0.169569 | 0.716449 | 0.513863 | 0.998902 | 0.049247 |
| AL136295.7 | 0.007657 | -0.1967 | 0.093299 | 0.821434 | 0.684157 | 0.986254 | 0.035004 |
| AC004975.2 | 0.043179 | -0.41689 | 0.188883 | 0.659092 | 0.455164 | 0.954385 | 0.027304 |
| AL136368.1 | 0.002268 | -0.66587 | 0.237609 | 0.513825 | 0.322524 | 0.818593 | 0.005073 |

HR Hazard Ratio, SE Standard error, B Regression coefficients, KM Kplan-Meier.
